# Testing the psychological pain hypothesis for postnatal depression: Reproductive success versus evidence of design

**DOI:** 10.1093/emph/eow032

**Published:** 2017-01-10

**Authors:** Edward H. Hagen, Randy Thornhill

**Affiliations:** 1 Department of Anthropology, Washington State University, 14204 NE Salmon Creek Avenue, Vancouver, WA 98686-9600, USA;; 2 Department of Biology, The University of New Mexico, MSC03 2020, 1 University of New Mexico, Albuquerque, NM 87131-0001, USA

## To the Editor

Myers *et al.* [1], in their interesting retrospective study of the association of postnatal depression (PND) with reproductive success (RS), conclude that their results ‘for the most part’, were ‘not supportive of the adaptive explanations’ proposed by us [2, 3] and others. Myers *et al.* did not test our adaptationist hypothesis for PND, however, but instead tested something else. Here we briefly summarize our adaptationist psychological pain hypothesis (PPH) and clarify how it differs from the adaptive hypothesis tested by Myers *et al.* Myers *et al.*'s results support the PPH. At the Editor's request, we also respond to Nettle's critique of the PPH [4] for PND and major depression (MD).

Under the PPH, a complex PND-mechanism evolved by sustained positive selection in ancestral environments because it helped optimize offspring quantity–quality trade-offs by reducing investment in offspring that were unlikely to survive or reproduce, which allowed mothers to invest more in existing or future offspring. In addition, the mechanism facilitated learning that would improve future decisions about childbearing and investment. This mechanism is now fixed in the population. All women inherit this PND-mechanism, but only the minority who experience socioecological conditions that ancestrally were not conducive to investing in a new child, such as lack of paternal investment or access to resources, or poor infant or maternal health (which we refer to as ‘poor maternal condition’), suffer PND symptoms [2, 3]. Myers *et al.* reported no measures of offspring quality, maternal investment, learning, or reproductive decision-making, and were therefore unable to test most aspects of the PPH.

Instead, Myers *et al.* appear to have operationalized an ‘adaptive’ hypothesis (which they disfavor) as follows: some women have inherited a propensity for PND and others have not, and, in a contemporary population (mostly women from the UK and US), mothers who experience PND will experience an increase in completed fertility, at least if they are in poor condition. For instance, they say ‘If PND is an aid to maternal investment decision making…then women in poor circumstances who have PND may be expected to benefit from future reproduction enabled by resources saved or gained from kin, *relative to those who do not experience PND*’ (emphasis ours). Correspondingly, their regression models investigate trait and trait×condition effects on completed fertility. PND×condition interaction terms, in particular, test if the effect of PND on fertility differs in mothers in good versus poor condition, which suggests that according to this adaptive hypothesis, and unlike the PPH, PND is expressed in mothers in both good and poor condition. These models are appropriate for testing whether a trait that is not fixed in the population and is expressed in mothers in both good and poor condition, is currently experiencing positive natural selection, albeit perhaps only for mothers in poor condition. (Under the PPH, we do not expect interaction effects between PND and poor condition because PND only activates in poor conditions.) See Table 1 for a comparison of both hypotheses.

**Table 1.** Comparison of the Myers *et al.* adaptive hypothesis for PND to the adaptationist psychological pain hypothesis

	Myers *et al.* adaptive hypothesis	Psychological pain hypothesis
Trait	PND	Mechanism that assesses maternal condition and generates PND
Expression of PND	Mothers with the trait, in both good and poor condition	Only when the mechanism detects poor condition and generates PND
Manifestation of fitness benefit	Contemporary UK and US	Ancestral environments
Variable or fixed in the population	Variable	Fixed
Implication of reduced parity progression among women with PND	Evidence for reduced fitness of women with PND trait	Possible evidence for a functional reduction in parental investment by women in poor condition, and/or a poor environment

Myers *et al.*'s main result was that, compared to women who did not experience PND, women who experienced PND at their first or second birth had lower completed fertility, primarily by not having a third child. They also found little evidence that PND was associated with higher completed fertility for mothers in poor condition (i.e. they found very few significant positive interaction effects). Under the Myers *et al.* hypothesis, PND as a heritable trait that is relatively insensitive to environmental conditions would currently be experiencing negative natural selection in this population, even among women in poor condition, and would thus not be adaptive. We agree.

Under our PPH, however, these results have a very different interpretation that supports the PPH. Although the PPH emphasizes reduced investment in the new infant, Hagen [3] noted that this was not the only way to reduce parental investment. Another way would be to limit completed family size. On this view, Myers *et al.*'s main result can be reinterpreted as evidence for a maternal decision-making mechanism that would have increased fitness in ancestral environments by limiting family size in poor circumstances, allowing increased investment in existing offspring (numerous studies support the association of PND with poor maternal condition [2, 3, 5]; indeed, that is why Myers *et al.* included several maternal condition variables in their study).

Another interpretation of the Myers *et al.* result that is consistent with the PPH is that if mothers with PND are in poor condition at, e.g. birth 1, then they are also likely to be in poor condition in the future, and it is this poor condition, and not PND, that limits family size. We tested if poor condition at birth 1 predicted poor condition at birth 2 and 3 by computing a ‘poor maternal condition’ score for births 1, 2 and 3 that was identical to Myers *et al.*'s (the sum of: birth complications, no breastfeeding, abnormal birth weight, infant health issues, and low father, family, and friend support) except that it excluded SES and support from the mother's mother, which did not vary between births, and poor birth emotions, which is confounded with PND. The poor maternal condition score for each birth was highly significantly correlated with that for every other birth (Table 2). Of these risk factors, we found evidence that birth complications reduced the probability of parity progression.

**Table 2.** Correlation matrix of poor maternal condition scores at births 1, 2 and 3

	Birth 1	Birth 2	Birth 3
Birth 1	1.00	0.54	0.48
Birth 2	0.54	1.00	0.60
Birth 3	0.48	0.60	1.00

*P* < 0.001for each correlation.

It is worth explaining why heritable variation and differential reproduction, despite being the basis of evolution by natural selection, cannot identify complex adaptations (those based on many genes). Complex adaptations are the product of a lengthy iterative process during which numerous simple traits arise via mutation and then go to fixation under natural selection. Once traits are fixed, variation in RS cannot be ascribed to a non-existent heritable variation in the traits. Using technologies like gene knockouts or surgical removal it is possible to compare the fertility of laboratory organisms with a complex trait to those in identical socioecological conditions that lack the trait. Even so, such tests can only show that the trait is important to reproduction; they cannot identify the function of the trait. In any case, it would be very difficult to conduct such tests in humans because the only individuals lacking the trait would be those whose mechanism was disabled due to, e.g. rare mutations, developmental disruptions, or pharmacological suppression. It is unlikely that Myers *et al.*'s online sample of 306 postmenopausal women, recruited via advertising newsletters and social media, would contain many, if any, such women. How, then, to test the PPH?

Complex adaptations are identified by evidence of functional design. Darwin formulated the theory of natural selection precisely to explain how species ‘acquire that perfection of structure and coadaptation which most justly excites our admiration’ [6]. George Williams developed this theme in his hugely influential book *Adaptation and Natural Selection*, where he criticized views that focused ‘attention on the rather trivial problem of the degree to which an organism actually achieves reproductive survival. The central biological problem is not survival as such, but design for survival’ [7]. We, too, have written extensively on the importance of functional design in studying complex adaptations, e.g. [8, 9].

Under the PPH the function of PND—its ancestral fitness benefit—was to optimize maternal investment in ancestral environments relative to maternal condition, and to promote learning. Our publications [2, 3] marshal the evidence that PND shows evidence of design to reduce parental investment when mothers are in poor condition. Future investigations of the PPH must more precisely specify maternal strategies for socioecological assessment, offspring investment, and learning, and then determine if PND shows evidence that it is well-designed to fulfill these functions.

Alternatively, researchers wishing to test the hypothesis that PND is an illness should employ Wakefield's *harmful dysfunction* illness concept [10]: illnesses are (i) dysfunctions of evolved adaptations (ii) that are harmful. A *dysfunction* is a perturbation that hinders or prevents an adaptation from performing its evolved biological function. *Harm*, on the other hand, is socially or culturally defined. We do not dispute that the symptoms of PND, such as reduced care and love of a newborn, are harmful. But many adaptations are harmful, including anger, aggression and jealousy. In fact, we suspect mainstream PND researchers conflate harm with dysfunction. To test if PND is an illness, one must specify the biological function/s that is/are dysfunctional. What adaptations govern maternal psychological/behavioral responses to poor postnatal condition, and what, exactly is going wrong in PND?

Despite much evidence in favor of the PPH, it is far from proven. Nettle [4], in particular, argued that PND and MD do not show evidence of good functional design. We largely agree with Nettle's criteria, most of which are also noted by Myers *et al.* We disagree, however, that current evidence against good design in PND outweighs the evidence in favor.

For instance, as both Myers *et al.* and Nettle [4] note, most women who experience a risk factor do not get depressed, and some who do not experience any risk factors still get depressed. We do not dispute this pattern, which represents the core mystery of depression, but it must be put in context. Depression scores, including the EPDS scores that were one of the PND measures used by Myers *et al.*, occur on a continuum, and depressed status is determined simply by exceeding a threshold score. The EPDS scale ranges from 0 to 30, and Myers *et al.* used a threshold of 12 for PND status. However, women with an EPDS scores of, e.g. 10 or 11 are still suffering psychological pain, and higher scores indicate more intense pain with no obvious shift to a qualitatively different psychological state. Moreover, these scores are proportionate to maternal condition, which we illustrate using the same risk factors used to compute Table 2, plus low SES and low support from the mother's mother. Complete data was available for 642 births. For the 102 births in which no risk factors were present the mean EPDS score was 5.9; for the 50 births with 5 or more risk factors, the mean EPDS score increased to 11.1. The increase in EPDS scores with increasing adversity supports the PPH.

Contrary to the PPH, there is considerable variation in EPDS scores for exposure to any given number of risk factors. For example, for the 73 births with 4 risk factors, EPDS scores ranged from 0 to 27, with a mean of 9.9 and an SD of 6.6. However, much of this unexplained variation could be due to inaccurate measurement of maternal condition. Myers *et al.* measured access to resources with SES estimated via broad occupational categories, for instance, which could miss important sources of variation in access to resources. Myers *et al.* also did not include all known risk factors for PND, such as unintended pregnancy [11] and domestic abuse and other forms of violence [12]. It is also almost certain that we do not yet fully understand human ‘maternal condition’, and that currently unknown dimensions of this construct help explain variation in levels of PND. And it is possible that, just like the immune system, the PND-mechanism sometimes activates when there is no adversity, or fails to activate when there is.

A second challenge is the evidence that PND is heritable, with heritability estimates ranging from 25 to 54% [13, 14], which as Nettle [4] pointed out, undermines the claim that a PND-mechanism is fixed in the population. There are three reasons why these estimates do not (necessarily) refute the putative universality of a PND-mechanism. First, this range of heritability estimates is considerably smaller than the heritabilities of bipolar I disorder and schizophrenia estimated from twin studies, which are >80% [15, 16]. Second, some of the heritability of depression appears to be heritability in stress sensitivity [17], and not in the ability to experience depression. Third, and perhaps most importantly, much of the heritability of depression appears to be heritability in exposure to environmental risk factors, i.e. genetic control of exposure to environmental adversity, and not in the ability to experience depression per se. In one large twin study of MD, the direct path from genetic risk for MD to an episode of MD in the last year was small, with most of the genetic risk operating indirectly via environmental factors, such as disturbed family environment and childhood parental loss [18]. In a review of 55 studies on genetic influences on the likelihood of experiencing 35 environmental risk factors, Kendler and Baker [19] conclude (p. 624):

[S]tandard heritability estimates cannot discriminate between inside and outside the skin pathways. Our results suggest that a non-trivial proportion of genetic effects assessed by twin and adoption studies for psychiatric and substance use disorders may involve selection into environmental adversity that then feeds back to increase disease risk.

In the case of PND, maternal condition could be heritable, for example, rather than psychological pain itself.

A third challenge to the PPH raised by Myers *et al.* and Nettle [4] is that depression in general, and PND in particular, appear to be chronic conditions, i.e. they do not remit when conditions improve, contrary to the PPH. However, much of the evidence for chronicity comes from selected clinical populations (i.e. patients who have sought treatment for depression), and not more representative community populations where the recurrence rates are much lower. For instance, Myers *et al.* cite Vliegen *et al.* [20] who reviewed 23 longitudinal studies and found that whereas about 50% of postnatal women in clinical samples remained depressed throughout and beyond the first postnatal year, this was true of only about 30% of postnatal women in community samples. Critically, in addition to previous history of depression and personality-related vulnerability, the few studies with an appropriate design found that chronic PND was associated with repeated or chronic exposure to stressful life circumstances. In summary, PND is not chronic in most women in representative community populations, and those for whom it is are often exposed to chronic stressors, consistent with the PPH.

A fourth challenge is that depression can cause stressful life events (SLEs), a point emphasized by Myers *et al.* for PND, and which inspired Nettle [4] to question whether SLEs actually cause depression. If not, this would disprove the PPH. However, most studies have found bidirectional effects: SLEs do cause depression, which then causes more SLEs [21]. The causal role of SLEs on depression is most clearly seen when SLEs are divided into those that are influenced by the individual's own behavior, such as interpersonal problems, versus those that are independent of the individual's behavior, such as death of a loved one. The latter category of SLEs is a strong predictor of depression, indicating a causal effect of SLEs on depression [22]. (The ‘kindling effect’, whereby the association of SLEs and depression decreases as the number of depressive episodes increases, might be a statistical artifact [23].)

We are not surprised that PND causes interpersonal problems. The mother plays a uniquely important role in the care of newborns. If she unilaterally defects from investing in the infant, this creates enormous problems for the father and other family members, which would certainly exacerbate interpersonal problems.

A fifth challenge raised by Myers *et al.* is that PND is associated with inflammation and increased morbidity, which at first glance seems unrelated to psychological pain and optimizing quantity–quality trade-offs. Domestic abuse is common in the perinatal period, however (albeit less so than at other times), with most studies in high-income populations finding rates of 4–8% during pregnancy [12]. Domestic abuse is a potent risk factor for PND [12]. Under the PPH, this is hardly surprising given the profound implications of physical and emotional abuse for maternal condition and ability to raise offspring. Under the PPH, inflammation is associated with PND because mothers have either suffered physical assault or are at increased risk for it, a pattern also seen in depression more generally [24], and/or that infection is contributing to poor maternal condition.

Another cost noted by Myers *et al.* is that PND is associated with poor child development, although the effects are small-to-moderate [25, 26]. Under the PPH there are three potential explanations of these effects: (i) mothers with PND are reducing investment in the infant, as predicted by the PPH, so as to increase investment in other (or future) offspring; (ii) the infant was low quality, which caused PND, as predicted by the PPH (i.e. causation is in the reverse direction); and (iii) poor maternal condition, and not PND, causes poor child development.

Finally, Myers *et al.* and Nettle [4] question the signaling/bargaining extensions of the PPH [3, 27–29] that predict increased investment by social partners, on the grounds that PND and MD are associated with disrupted social relationships and often evoke hostility and rejection from social partners. Myers *et al.* and Nettle fail to acknowledge the central role of *conflict* in the signaling/bargaining models. Anger causes negative reactions in targeted social partners, yet it is probably an adaptation that yielded fitness benefits during social conflicts by exploiting advantages in physical or social formidability to force concessions from others [30]. Similarly, mothers who are *in conflict* with their husbands and other social partners can put the baby's health, and/or their own health, at risk to force concessions from unwilling fathers and other family members.

The evidence that maternal PND increases paternal investment is mixed, with some studies finding support, e.g. [31–33], and others not, e.g. [34–36]. Although the original versions of the bargaining model [3, 27] predicted that maternal PND should increase paternal investment, a later refinement [28], based on non-cooperative game theory models of bargaining, viewed depressive symptoms and reduced parental investment as a means for each parent to reveal their private valuations of the new offspring to the other parent and family members. On this view, fathers would not increase investment if they do not value the offspring. Future studies should employ a longitudinal design that tracks both parents' valuations of the child, their full range of depressive symptoms, and their levels of child investment from pregnancy through the postpartum period.

Nettle [4] claimed that there was no evidence that depression in general elicited positive reactions from social partners. This is incorrect. Social partners reduce aggression and increase positive reactions in response to depressive behaviors, so much so that many researchers worry these benefits reinforce depression [37–39]. Furthermore, in small kin-based societies, much (but not all) suicidal behavior, an important symptom of depression, is a response to conflict by powerless individuals that often elicits benefits if the victim survives [40].

The PPH would be falsified if there were substantial evidence against any of the following predictions: (i) PND symptoms should have relatively high sensitivity and specificity for poor maternal condition; (ii) PND symptom levels should be proportionate to maternal condition; (iii) PND symptoms should alter parental investment in ways that would have optimized quantity–quality trade-offs in ancestral human environments; (iv) PND should activate learning and other mechanisms that, in ancestral environments, would have improved maternal condition; and (v) the PND-mechanism itself should have low heritability.

In broader perspective, there might be fewer differences between our view and those of Myers *et al.* and Nettle than it seems. Myers *et al.* and Nettle describe PND and MD as syndromes that have little association with SLEs and have high relapse rates resulting in long term chronic disability, which characterizes these phenomena in clinical populations but much less so in representative community populations, where PND and MD for the most part are linked to SLEs, symptom levels are proportionate to levels of adversity, and only a minority suffer a second episode. Horwitz and Wakefield [41] argue that when the Feighner and Research Diagnostic Criteria, which were developed to distinguish mental disorders *within* clinical populations, became the basis for DSM-III and were (mis)applied to community populations, they generated a substantial excess of false positives for disordered sadness, primarily because these criteria completely ignore life circumstances (the only exception being the ‘bereavement exclusion’, which was eliminated in DSM-V). The PPH applies to the full range of PND symptoms in representative community populations, not primarily to suprathreshold symptoms in clinical populations.

We suspect that Myers *et al.* and Nettle would also agree with us that because PND is harmful (though not necessarily dysfunctional), it is important to devise social policies that help prevent it by ensuring that all pregnancies are intended and all mothers are in good perinatal condition and able to ‘afford’ the new infant.

**Conflict of interest**: None declared.
